# Deficient editorial practices, perceived quality, and expedient scholarly publishing in a developing nation

**DOI:** 10.12688/f1000research.134583.2

**Published:** 2023-11-30

**Authors:** Amin Bredan, Osama Tashani, Omran Bakoush

**Affiliations:** 1 Independent researcher, Ghent, Flanders, Belgium; 2 MENA Research Group, Leeds Beckett University, Leeds, England, UK; 3 College of Medicine and Health Sciences, United Arab Emirates University, Al Ain, Abu Dhabi, 15551, United Arab Emirates

**Keywords:** Citation counts; Journal affiliation; scholarly publishing; Science editors; Libya

## Abstract

**Background:**

There is increasing concern about the quality, integrity, and accessibility to research published in the developing world. This study explores the editorial practices and editors’ perspectives to gain insight into the standard of scholarly publishing in Libya.

**Methods:**

Between 21 ^st^ January and 12 ^th^ February, 2022, the editors-in-chief (EC) of Libyan academic journals were invited to complete a questionnaire on editorial practices, degree of satisfaction with submitted and published manuscripts, review processes, and journal performance, as well as challenges facing the journals. Journal websites were examined for quality, and indexation coverage and citations were assessed. We examined the number of citations in Google Scholar for all 2019 articles published in each journal. Descriptive statistics were used to quantitatively summarize the data and thematic analysis was used for the narrative text.

**Results:**

48 EC completed the questionnaire. The EC was affiliated with the institution that owns the journal in 92% of cases. Most EC (83%) were satisfied with the peer-review quality, 69% believed that most of their published papers add new ideas or findings, and 96% were satisfied with their journal’s performance. However, despite the high degree of satisfaction, only one journal was indexed in Web of Science or Scopus and only 17% of the journals were indexed in Google Scholar. A qualitative assessment of journal websites revealed shortcomings in publishing practices in a large proportion of the journals.

**Conclusions:**

The discordance between the satisfaction of the journal editors and the journal quality indicators points to a break in the quality system of Libyan academic publishing. Similar expedient publishing practices might exist in other countries as well. A comprehensive action plan led by academic institutions to enforce high standards for scholarly publishing is needed to advance research and high-quality scholarly publications in developing countries.

## Introduction

Quality scholarly publications are known as the most effective means of disseminating locally produced scientific research findings to the global scientific community. However, there is increasing concern about the quality, integrity, and accessibility to research published locally. ^1^ Journals may become poor venues for knowledge dissemination if they fail to provide adequate peer review, quality control, and content preservation. Researchers from developing countries face the challenges of generating sufficient high-quality research and sharing their research findings due to academic, technological, socio-political, and economic factors. ^2^

Libya is an Arab country with an estimated surface area of 1,775,500 km ^2^ and a population of about 6.7 million. Most of the population live in the main coastal cities. Since the start of oil exportation from Libya in the 1960s, living standards have risen. Undergraduate and postgraduate institutions have been expanded and improved over the years. The first Libyan university was established in 1957 ( https://mhesr.gov.ly/?page_id=126). The number of universities has expanded over time, and according to the ministry of education, there are now 26 public universities in Libya with 350,000 university students. ^3^

Many developing countries have increased their rates of contribution to the world’s scientific output, measured as the number of publications and the number of citations. ^4^ However, Libya maintained low scholarly publishing rates when compared to other countries in the region, including those with considerably lower economic indices. ^5^ The first initiative to formally promote research in Libya began in the mid-seventies with the establishment of the National Commission of Scientific Research, formally charged with planning and supporting scientific research. Over many years, the Libyan government expanded the scope of MSc degree programs, and recently initiated PhD programs in various fields, including sciences, as well as built research centers. However, overall research performance indicators remain modest.

The first scholarly journal published in Libya, “Al-Majalla Al-Libiyah Lil-‘Ulum” (The Libyan Journal of Science), was launched in 1971. It is improbable that it is the currently active journal by the same name because the numbering of the annual volumes goes back only to the late nineties. Jamahiriya Medical Journal was launched in 1976 and continues to be listed in Scopus (without metrics) though it ceased its activity in 2011. A recent bibliometric study ^6^ based on the Scopus database for Libya’s scientific output from 1948 to 2022 revealed a total of 10,475 articles, 10361 of them in English (99%). On the other hand, another study, ^7^ based on the Directory and Book of Abstracts of Libya’s National Centre for Scientific Research for 2001-2004, revealed that 45% of the research output was reported in English, and the rest was in Arabic.

The quality of journals is intimately linked to the quality of editorial practices. Guidelines have been published by the Committee on Publication Ethics (COPE) ^8^ and the Council of Science Editors (CSE). ^9^ Guidelines have also been published for medical journals by the World Association of Medical Editors (WAME) ^10^ and the International Committee of Medical Journal Editors (ICMJE). ^11^ The regional association Forum for African Medical Editors (FAME) ^12^ also provides support and resources for best editorial practices. The major abstract-indexing databases of Web of Science ^13^ and Elsevier’s Scopus ^14^ specify criteria required for indexation. Publishing research papers in journals that do not adhere to editorial best practices is a waste of research funds and findings. Moreover, poor-quality journals could lead to a decrease in the quality of science at both the institutional and national levels, with university staff and research scientists getting recognized without gaining the required research expertise, including in publishing ethics and research integrity. Such a situation could have a negative impact on the academic environment and on researchers’ aspirations and practices when conducting research. The effects of all that could filter down to university and graduate students, and beyond.

Gaining an understanding of the status and functioning of local, national, academic journals would shed light not only on their editorial quality, but could also indicate the areas that need improvement in order to raise the country’s research quality and performance. This study aimed to explore the editorial practices of editors related to scholarly publishing in Libya and quality indicators of the academic journals. We also sought the editors’ perspectives on the standards of academic publishing in Libya and their satisfaction with journal performance.

## Methods

### Ethical statement

Ethical approval for the main research project “Publishing practices in scholarly journals in Middle East and North Africa” was obtained from UAEU Social Sciences Research Ethics Committee (ERS_2021_8420). The questionnaire was prefaced with a description of the study aim, declaration of ethics approval, statement of the voluntary nature of participation, and the ability to withdraw. Consent was signaled by the answer to the first question.

### Study design

This study was carried out between January 21 ^st^ and February 12 ^th^, 2022. Our target consisted of the editors-in-chief (EC) of academic journals affiliated with Libyan academic institutions and professional societies. Online survey links were sent by the primary investigator (AB) via the editors’ contact emails identified in the journal websites. Participants received a standard follow-up email reminder two weeks after they received their original invitation to participate.

We designed a questionnaire consisting of 10 parts, following the best practices in designing questionnaire items and organizing the questionnaire. ^15^^,^^16^ The questionnaire was administered in English and Arabic and distributed using SurveyMonkey. ^17^ The themes of the questions were guided by the editorial policies published by the CSE ^9^ and the basic journal selection criteria for indexation of the Web of Science. ^13^ The questionnaire was reviewed independently by three editors of an international journal who were not affiliated with a local or regional institution. They were asked to assess the face validity of the survey items and to provide feedback on the clarity and conciseness of the questions. No ambiguous questions or other issues were identified in the questionnaire and therefore no changes were made.

We then posted the questionnaire using an online survey program, SurveyMonkey ( https://www.surveymonkey.com/), which allows respondents to answer the questions but does not allow duplicate responses. The link to the online survey and the cover letter were then sent by electronic mail to the EC. A reminder was sent after two weeks to those who had not responded. In total, 51 questionnaires were returned. The responses were downloaded as a Microsoft Excel file (2007). Descriptive and inferential statistical analysis was done in SPSS statistical software (version 26.0, IBM). PSPP ( https://www.gnu.org/software/pspp/) is a freely accessible software application that can run the same analysis used in this study.

The 10 parts of the questionnaire were as follows:

1. The journal’s current status;
2. Background questions about the editors’ affiliations;
3. Editor satisfaction;
4. The editorial process;
5. The peer review process;
6. Satisfaction with peer review reports;
7. Journal indexing;
8. Journal funding and support;
9. Editorial independence;
10. Challenges and opportunities.

To enhance the trustworthiness of the interpretations from the survey quantitative data, a word cloud was generated of all the words in all the respondents’ answers to the question about challenges faced by the journals, after translating Arabic text to English. The entire text was analyzed in an online word cloud generator ( https://monkeylearn.com/word-cloud).

To integrate the results of desk research with the results from the questionnaire, the websites of the journals the editors of which returned questionnaire responses were visited by one investigator (AB) and information was collected on various journal qualities. These included but were not limited to the presence of an International Standard Serial Number (ISSN), the editorial board’s geographic diversity, the organization of the archives, and general website functionality. Moreover, data were collected on the listing of the journals in Google Scholar and the number of citations of all articles published in 2019, as well as indexation in the database of the Arab Citation & Impact Factor (ARCIF).

### Statistical analysis

The coded data were transferred to SPSS and analyzed by another investigator (OT). The results were analyzed by the percentage of participants selecting the different answers to each question. The 95% confidence intervals are given as ranges inside brackets. The responses to the two open-ended questions about challenges and opportunities were analyzed by thematic analysis. The journal quality assessment and Google citations are reported as frequency and percentage statistics.

## Results

### Questionnaire results

In total, 51 questionnaires were returned. However, three responses were excluded because the journals did not have the basic content of a published academic journal, such as an official website. Therefore, the study included 48 academic journals published by national institutions (list available at https://10.5281/zenodo.10155318). Some EC skipped answering some questions, but their available answers were included in the analysis.

*Journal ownership*

40 of the 48 journals are owned by academic institutions (83%, 95% CI: 70–93%). The others are owned by a professional society, a professional syndicate, or a research center.

*Editorial boards*

The EC was affiliated with the institution that owns the journal in 44 out of 48 journals (92%, 95%CI 80–98%). In 29 journals (60%, 45–74%), all the editors other than the editor-in-chief were associated with the owning institution. In nine journals (19%, 9–33%), the editors were associated with various national institutions, and 10 journals (21%, 10–35%) have a mix of nationally and internationally affiliated editors.

*Journal funding*

Three journals did not respond to the question about journal funding. 32 of the 45 responding journals (71%, 95%CI 56–84%) are supported by the institution that owns the journal, and 22% (10–37%) by author processing charges (APCs). Professional societies played a minor role (3/45, 7%). In the 35 journals supported by the journal owner, the support covered the website cost of 27 journals (77%, 60–90%), software for journal management or similarity check of six journals (17%, 7–34%) and financial rewards for editors and/or reviewers of 14 journals (40%, 24–58%).

*Peer review process*

**Figure 1.**
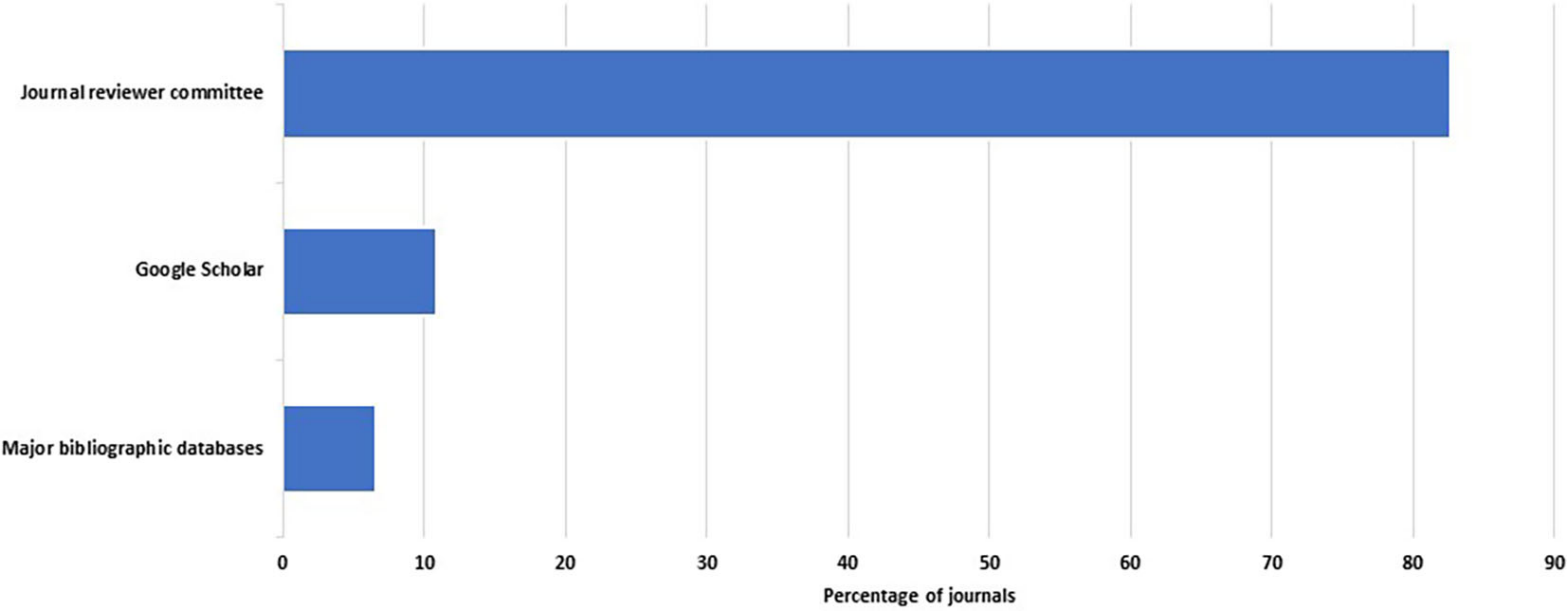
Percent distribution of sources of reviewers.

**Figure 2.**
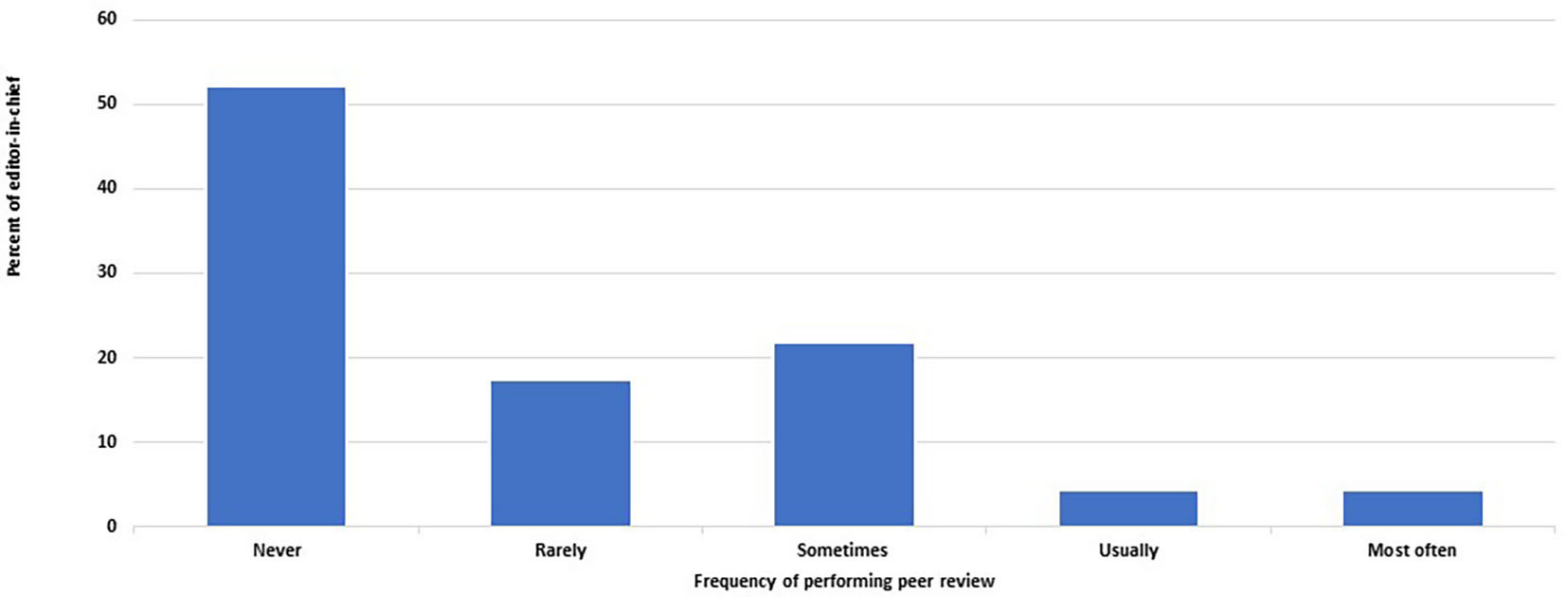
Frequency of editors-in-chief who perform peer review of submitted manuscripts and write reports.

A total of 46 of the 48 journals described their peer review process. Reviewers are recruited within two weeks in 31 of 46 journals (67%, 52–80%). Many or all peer reviews are done by individuals who are not members of the editorial board in 85% of the journals (95% CI: 71–94%). However, there was heavy reliance on the journals’ reviewer committees. These committees were the main source of reviewers in 38 of the 46 journals that responded to this question (83%, 69– 92%) ( Figure 1). The EC performs peer review and writes reports with various frequencies in 22 out of 46 journals (48%, 33– 63%) ( Figure 2). On the other hand, editors other than the EC never undertake peer review or do so rarely in 26 of the 46 journals (57%, 41–71%).

With two EC abstaining, 11 of the 46 responding EC (24%, 13–39%) said that they implement a similarity check against the Crossref database using iThenticate plagiarism checker, four (9%, 2.4–21%) use online programs (not specified), and 22 (48%, 33–63%) use Google. Nine (20%, 9–34%) do not perform any check for similarity against the published literature.

Only 10 journals (22%, 11–36%) use an electronic journal management system to communicate with authors and reviewers. The most prevalent communication medium was email (n = 35, 76%, 61–87%). Interestingly, in one case, communication was by ‘personal contact’.

The minimum number of reviewer reports required to make a decision was two or three in 41 of the 46 responding journals (89%, 76–96%). Four journals required only one, and one journal reported no reviewers.

*Acceptance and rejection rates*

**Figure 3.**
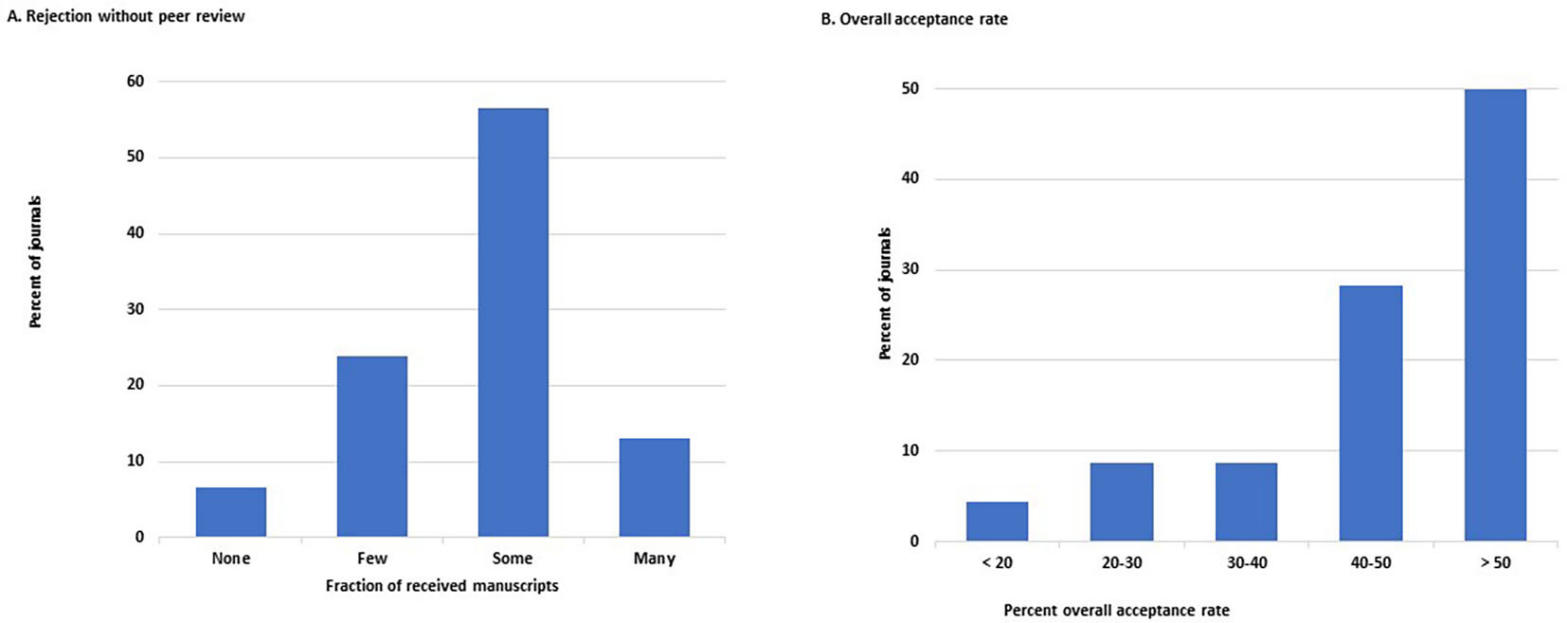
Distribution of the journals by frequency of manuscripts rejected without peer review (A) and by overall percent acceptance rate of submitted manuscripts (B).

Over half of the 46 journals that answered this question (26/46, 57%, 95%CI: 18–48%) reject ‘some’ manuscripts at the initial stage of editorial evaluation, whereas 14 of the 46 journals (30%, 18–46%) reject only ‘a few’ or none of them without peer review. Only six journals (13%, 5–26%) reject ‘many’ ( Figure 3A).

The overall acceptance rate of all received manuscripts was >40% in 36 of the 46 journals that responded to this question (78%, 64–89%). On the other hand, the acceptance rate was <20% in only two journals (4.3%, 0.5–15%) ( Figure 3B). No journal had an acceptance rate <10%.

*Proof-reading of published manuscripts*

Most journals proof-read Arabic and/or English manuscripts. However, one-quarter of them (24%, 13–39%) do not proof-read the articles before publication.

*Indexing in abstract and citation databases*

In asking about inclusion of the journals in abstract and citation databases, we gave Web of Science and/or Scopus as one choice, and as another, Arabic Citation Index (ARCI), a new index for scholarly publications in Arabic established by collaboration between Clarivate Analytics and the Egyptian Knowledge Bank and funded by the Egyptian government. Four journals did not provide information on indexing in databases. Of the 44 responding journals, 20 (45%, 30–61%) stated that they were not in any such database. One journal was verified to be indexed in Scopus and Web of Science. 10 journals described as indexed in ARCI could not be verified due to the unavailability of a publicly available ARCI journal list. It is possible that the EC confused ARCI with ARCIF.

Of the 32 editors who responded to the question about submission of their journals to an index, 30 (94%) answered that they had submitted. Four journals (13%, 4–29%) had been submitted to Web of Science or Scopus, but the entities to which the other journals had been submitted were not disclosed. Notably, 21 of 23 EC (91.3%, 72–99%) cited lack of funding as an impediment to submission to abstract-indexing databases.

*Assessment of satisfaction with the journals*

Satisfaction with the journal’s performance

**Figure 4.**
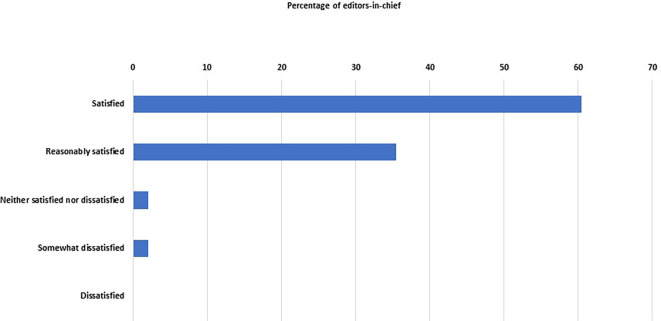
Percentage distribution of the degree of satisfaction of the editors-in-chief with the performance of their journals.

We asked the EC about their satisfaction with their journals’ ‘performance’, and 46 of them (96%, 86–99%) were either satisfied (n = 29) or reasonably satisfied (n = 17) ( Figure 4). Only two EC were not satisfied.

**Figure 5.**
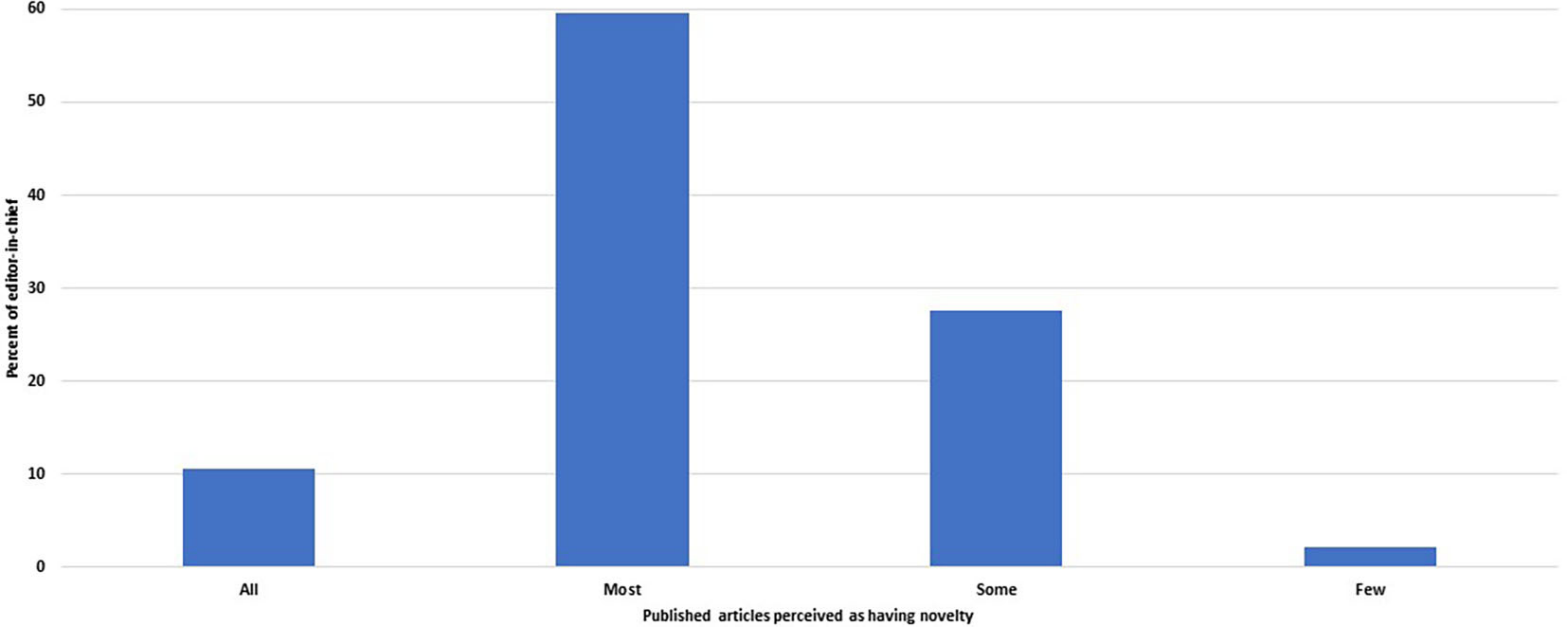
Distribution of the journals by the fraction of published articles perceived by the editors-in-chief as adding new findings or ideas.

Of 48 editors, 33 (69%, 54–81%) believed that all or most of the papers they publish add new ideas or new research findings ( Figure 5). The other 29% (17–44%) felt that only some papers had novelty, and one EC did not know.

Satisfaction with the manuscripts submitted to peer review

**Figure 6.**
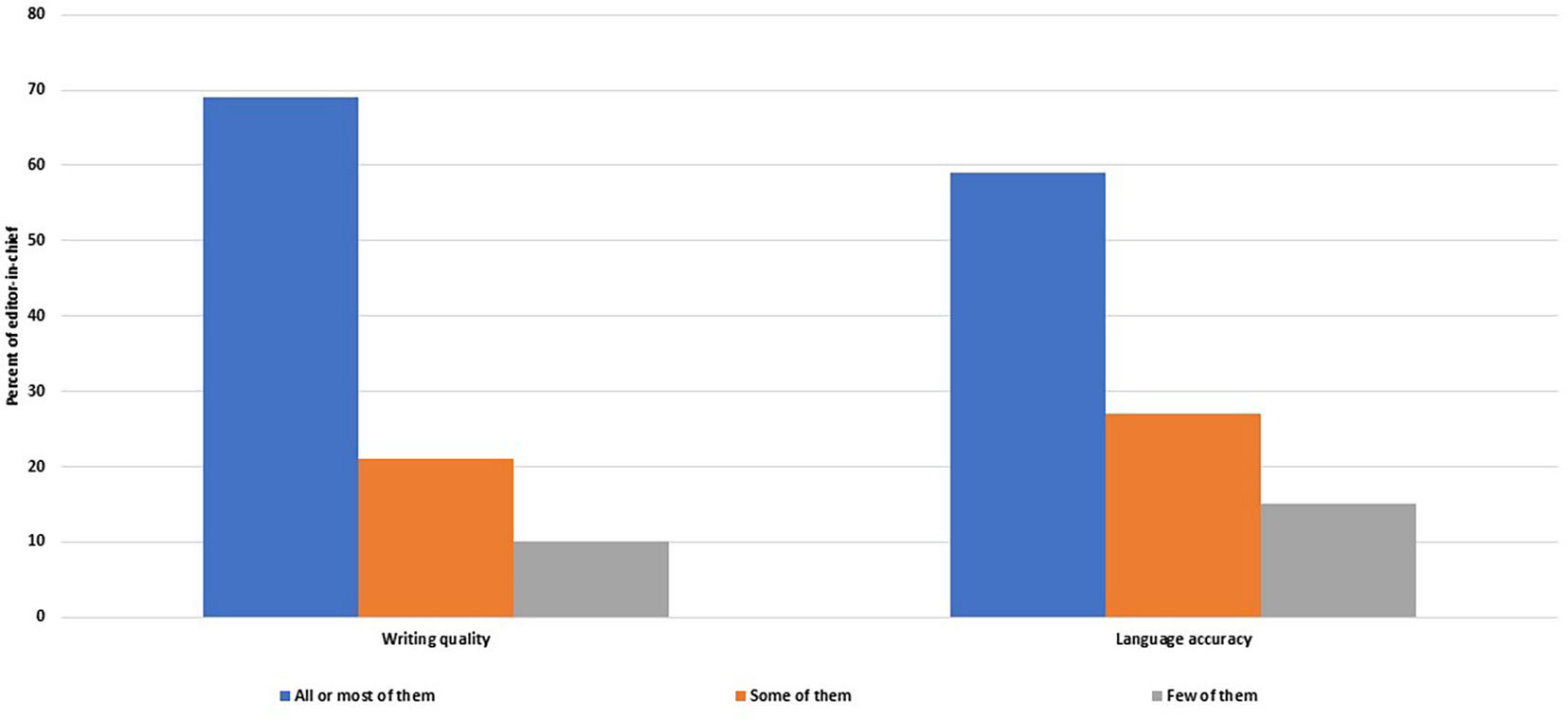
Frequency of manuscripts sent to peer review that satisfy the editors-in-chiefs in terms of writing quality and language accuracy.

Most of the EC (n = 33, 69%, 54–81%) were satisfied with the quality, clarity and organization of most or all of the manuscripts submitted to peer review, and 28 of them (58%, 43–72%) were satisfied with the accuracy of the language of all or most peer reviewed manuscripts ( Figure-6).

Satisfaction with the quality of peer review reports

A total of 46 EC answered the question about satisfaction with reviewer reports. A large majority (38, 83%, 69–92%) were satisfied most of the time or always. 42 of them provided the reasons for their satisfaction. Roughly half of them were satisfied with comprehensiveness and thoroughness of the reports, the questions they raise, and/or the suggestions and helpful comments they provide ( Table 1). The lowest frequencies of satisfaction were with the punctuality of the reports (24%) and inclusion of comments on the novelty and significance of the research (26%). Four editors gave the reasons for their dissatisfaction with the reviewer reports, two of whom mentioned the absence of all five reasons for satisfaction listed in Table 1.

**Table 1.** Reasons for satisfaction of the editors-in-chief with the reviewers' reports.

Reasons for satisfaction with reviewer reports	n	%
They are usually comprehensive, thorough, objective and insightful.	23	55
They usually raise questions about the study and offer constructive suggestions to be addressed by the authors.	20	48
They usually include comments about the study's novelty & importance.	11	26
They usually include helpful comments to the author and the editor.	24	57
They are usually submitted early or on time.	10	24

n = 46; Two journals no response. Multiple answers were allowed.

*Attempts to influence editorial decisions*

Attempts by superiors or the institutions to influence editorial decisions were described as rare by 96% of the EC and as ‘sometimes’ by the others.

*Challenges and opportunities*

At the end of the questionnaire, the participants were asked to express their views on the challenges they face in the quality of journal publishing. 36 EC provided responses. Thematic analysis of the answers revealed two themes. One is the lack of financial and institutional support, exemplified by what one participant wrote:

“The most prominent limitation is the lack of support, even from the university, which the Journal is supposed to represent, not to mention the difficulties of the publishing process.” (#28)

Another participant was more specific about the financial impact on the publishing process:

“[the challenges we face include] unavailability of financial support from the parent institution. … .. Failure to provide basic programs such as the scientific theft detection program. Failure to release members of the editorial board to perform their duties to the fullest. The prevailing custom now is [this is] a voluntary work and they give you 300 Libyan Dinars every six months, which is not even enough to cover internet expenses. Non-disbursement or awards to reviewers.” (#36)

The second theme identified concerns the reviewing process and a clear dissatisfaction of the editors with the quality and quantity of manuscripts submitted. One participant wrote the following:

“[the main challenge in my opinion is] the reviewing process, [lack of] scientific quality and language quality of the submitted manuscripts.” (#24)

These two themes were again identified when the participants were asked to express their opinions on the opportunities available to overcome the challenges facing the journal publishing quality. They overwhelmingly suggested that financial and institutional (governmental or otherwise) is needed to overcome several challenges. In addition, there was a trend in the participant responses pointing to the technical and academic training and know-how of the publishing process of the editorial staff and the institution. One participant discussed in his response the need for education within the institutions to raise awareness of the importance of scientific publishing:

“the state institutions, researchers, universities and research centers are required to understand the importance of scientific publishing to upgrade these institutions and to develop a national strategy that focuses on a specific number of the field, providing them with the necessary technical and financial support, and finding a clear mechanism for communication with international institutions interested in publishing and finding a clear mechanism to facilitate the payment of the required subscriptions.” (#2)

**Figure 7.**
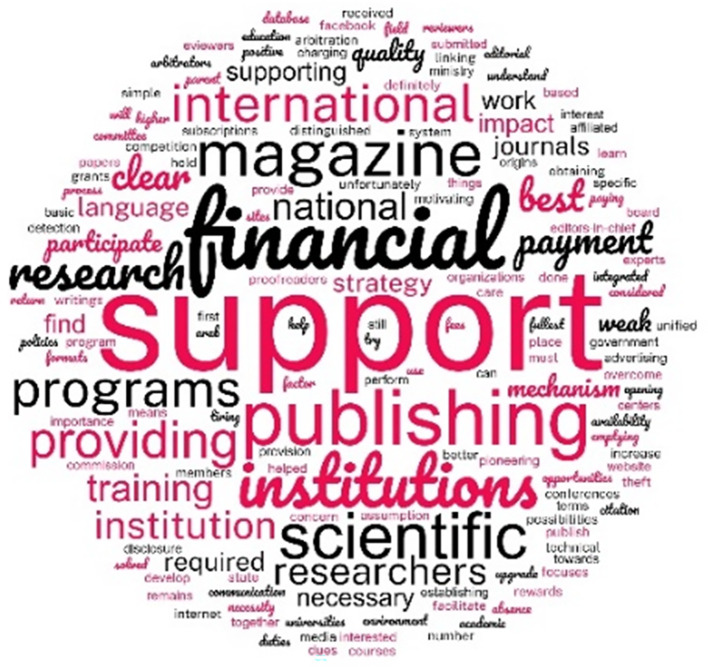
Word cloud of all the words in all the answers to the question “What are your opinions on the challenges facing the journal publishing quality?”

A word cloud ( Figure 7) of the answers to the question about the challenges faced by the journals shows that the EC view inadequate support and particularly financial support as the main challenge to raising the quality of the journals.

### Website-based journal assessment

Assessment of journals was made difficult by the inaccessibility of some journals’ websites or their content. Seemingly, this was caused by technical problems or updates or changes to the websites. One journal website was not accessible for review and the content of three other journals was not available for review. Thus, only 44 out of the 48 journals was accessible for this assessment.

*Scope, publication types, and language*

Overall, the journals cover a wide range of basic and applied sciences, social sciences and liberal arts, and some of them publish in a combination of different fields of science simultaneously, such as chemistry, medicine, botany, and information technology. Some journals publish articles in a wider variety of disciplines, including a combination of applied sciences and humanities.

According to the website information, in addition to publishing research articles and reviews, some journals publish conference proceedings and summaries of books, theses, dissertations, and reports on seminars.

*International Standard Serial Number (ISSN) and editorial board*

Examination of the journals’ websites showed only 30 journals displaying ISSN clearly on the website (75%, 59–87%) and in six journals (15%, 6–30%) it was embedded inside pdf files of entire issues consisting of up to hundreds of pages.

We examined whether the names of the editorial board members and their affiliations are mentioned. A total of 41 journals mentioned their editorial board members whether on the website as such or inside combined files containing entire issue or volumes. However only 17 journals mentioned details of their editors’ affiliations.

*Website organization and navigation*

A general qualitative assessment was made of the structure and functionality of the journal websites. The findings were extremely varied. Whereas some journal websites were professionally structured, organized and fully functional, including but not limited to the very few journals with professional publishers, the websites of a large number of journals had various types of defects, including broken links, disordered archives, missing essential information, and/or language mistakes. We also noticed that the secure socket layer certificate (SSL) was recently expired on a journal website, generating a security warning from the browser.

Some journals were difficult to navigate or required several clicks to reach the desired information. In one journal, three clicks are needed to arrive at the table of content of a selected issue, and then two more to download the article file. Random cases of a journal website going offline for a while were encountered. Some of these seem to have been during website update or maintenance, but no ‘maintenance’ page was used to temporarily substitute for the home page. We were unable to access the several journals of the second largest university over several months, and we received no response from the webmaster to our inquiry. Consequently, these journals are not included in this study.

Notably, a few journals advertised on their websites impact factors that are known as or suspected of being fake. Advertising a questionable impact factor can be detrimental to a journal, and any claim to having an impact factor should specify its source. When the source of a published impact factor is not specified, a deceiving intent to provide false impression about journal status is suspected.

*Journal archives*

Almost all the journals provide the articles only as pdf files. In some journals, some issues are missing or are not in proper chronological order, or the issues/volumes are numbered but do not mention the year.

13 journals (30%) publish their articles only as pdf files of entire issues containing many articles and reaching several hundred pages, most of which include journal information in the first several pages of the pdf files. Journal information of varied quality is in the first several pages of each pdf file in nine journals and on the website in two. The other two journals provide no information about the journal. Notably, four journals have repositories of separate pdf files for the articles, two of which do not provide any journal information anywhere, one gives it in a separate pdf file dedicated to journal information, and one has it on a website page.

*Policies*

Peer review policy

We considered that a clear statement about the peer review policy is adequate even if it is brief, providing that it mentions whether peer review is blinded and mentions the type of blinding. Of the reviewed journals, 15% specify double blind review, 17% describe the review only as being confidential, 48% simply state that the manuscripts are reviewed by experts, and 20% provide no information on peer review. One journal publishes the names of the members of the reviewer committee; there were only nine reviewers to cover multiple fields.

Open access policy

All the journals are full open access, of which only nine journals (20%) have a clear policy of either requiring no APC or stating the amount. Notably, some journals that have APC do not specify the amount but refer to it in vague ways that do not inspire confidence.

Human and animal study ethics

Based on the Helsinki Agreement, ^18^ the protocol for any study on animals or humans should be reviewed by an independent ethics committee (institutional review board), and in the case of humans, informed consent should be obtained from the participants in advance. An ethics statement was frequently absent in many journals. In some journals, none of the articles surveyed contained such a statement, while in other journals, it was included only in some articles. When ethics approval was obtained, it was not always from an ethics committee but from an administrative official. Moreover, when an ethics declaration was present, it did not always include both approval and consent. When consent was obtained, it was not stated whether it was oral or written.

### Indexation and citation analysis

As the results of the questionnaire showed that only one journal was indexed in Web of Science or Scopus, we searched for the journals in Google Scholar by using their names as ‘source’. Only eight journals (17%, 8–31%) were listed and the remaining 40 journals were not listed in Google Scholar. In the eight Google-indexed journals, half of the journals had international editorial boards, but in the unindexed journals only six (15%, 6–31%) had international editorial boards (RR 3.7, CI:1.12–12.25, p = 0.03). The difference is significant at p < 0.03.

As seven of the eight Google-indexed journals publish in fields of science, we compared their editorial board compositions with unindexed journals also publishing in sciences. Four of the seven indexed science journals (57%, 18–90%) had international editorial boards, whereas only three out of 13 unindexed journals (23%, 5–53%) had international boards (RR 2.5, CI: 0.76–8.07, p = 0.14).

We looked up the number of citations in Google Scholar for all articles published in 2019 in the eight journals indexed by Google Scholar. Three journals had no citations, and in all of them, all the editors were from the institution that owns the journal. In contrast, the editorial boards of four of the five cited journals (80%) were affiliated with a mixture of international and national institutions.

Among the cited journals, one had an article-citation rate of 90%, with an average of 5.8 citations per cited article, one had a rate of 40% with 1.2 citations per cited article, and three had rates in the range of 18–22%, with an average of 0.2–0.3 citations per article. One of the five journals is with a professional publisher, three use OJS, and the fifth uses a free open source content management system.

Only five journals’ DOIs (Digital Object Identifiers) and their metadata are deposited in Crossref database ( https://www.crossref.org/), and two other journals’ metadata are deposited in the Zenodo open repository ( https://zenodo.org/). None of the journals is in LENS or OpenAlex.

On the other hand, Marefa ( https://e-Marefa.net) is an abstract-indexing database established in 2008 for peer-reviewed literature from Arab countries published in Arabic, English or French. It indexes over 4000 journals and is the basis of the Arab Citation & Impact Factor (ARCIF, https://emarefa.net/ARCIF/). Of the 48 journals included in this study, 13 (27%) are listed in e-Marefa.com, with ARCIF factors ranging from zero to 1.3.

## Discussion

The gatekeeping role of high standard editorial practices is believed to be necessary to maintain the integrity of the scientific literature and minimize the risks of bad or low-quality science entering the scholarly record. Evidence-based publishing practices do benefit from scholarly communication, while journals with subpar standards may unprofessionally publish quantity rather than quality. Understanding what influences the quality of scholarly publications should be of the highest priority to academic institutions and policy-makers, especially given the consequences on the nation’s science advancement.

Many researchers who lack an appropriate funding system are unable to pay the large author publication fees charged by publishers, and the number of journals with good standing but without publication fees has been diminishing rapidly. ^19^ This pushes these researchers to publish in local journals. However, local journals should not be undervalued. Researchers might prefer to publish in international journals, but local journals have distinct, useful functions. Whereas international journals provide a platform for researchers to share their research globally, local journals provide a platform for issues and developments relevant to the local community that might not be considered relevant to international journals. Local journals also foster cooperation between local researchers and provide a platform for publishing in their native language. Nevertheless, there is a need for establishing robust criteria for publishing practices and policies of local journals, particularly university-based journals. This study provides an overview of the publishing practices in Libya and identifies several shortcomings. This study’s findings would be useful for establishing robust criteria to enhance trust in publishing practices and policies of journals affiliated with local institutions, particularly those that are not included in the major abstract-indexing databases.

The ISSN is an essential, unique identifier assigned by multiple centers around the world for a fee of only 25–50 euros ( https://portal.issn.org/sites/default/files/guidelinespublishers_merged-eng-final.pdf). Yet 15% of the journals did not have an ISSN and 18% of them did not display it on the website. Website display of a valid ISSN is a basic requirement of inclusion in Directory of Open Access Journals (DOAJ) ^20^ and other indexes.

The affiliations of the editorial boards are also not shown on many journal websites. This omission could have been due to lack of awareness, a suggestion that is supported by the other side of the coin, where two journals provided multi-page *curricula vitae* of the editors.

Proper functionality is important for any type of website. Awkward navigation, broken links, repeated downtime and language mistakes reflect negatively on the journal. Many of the journal websites we surveyed had such issues.

One notable though not surprising finding is the association between international composition of the editorial board and indexing in Google Scholar, and possibly with citation of journal articles in Google Scholar as well. The importance of an international editorial board is highlighted by Elsevier’s basic criteria for acceptance in Scopus, which includes geographic diversity of the editors. ^14^ However, geographic diversity should not be interpreted in a narrow sense, particularly in countries with small populations and tight social relations. More commonly, geographic diversity is understood to mean international diversity. An international editorial board can provide wider and more varied expertise, as well as greater editorial independence. Unfortunately, the criteria set by the committee appointed by the Libyan government for accreditation of Libyan scientific journals run counter to these precepts by dictating that the members of the editorial boards should be affiliated with the institution that owns the journal. A more effective approach for promoting the quality of the journals is to require or at least encourage international diversity of editorial boards.

A limited number of journals post the logos of indexes and impact factors that are bogus or at least have a bad reputation, and even display an ‘impact factor’ when they are not even indexed in Google Scholar. This is detrimental to the prospect of receiving submissions from serious scientists. In this study, the Google Scholar-cited journals do not do this. When an official body exists to oversee the quality of journals, this should be banned. The official committee mentioned in this report does not address this issue in its criteria.

In a study of acceptance rates, the overall acceptance rate in ‘ordinary’ open access publishers, including universities and societies, was about 50%. ^21^ According to the estimates of the EC in our study, the acceptance rate of all submitted manuscripts was more than 50% in half of the journals, and we speculate that some of them have much higher acceptance rates. Notably, of the 14 journals that rejected none or a few manuscripts without peer review, seven of them had an overall acceptance rate of > 50% and four had a rate of 40–50%.

The EC expressed a high degree of satisfaction with the performance of their journals, but this is not reflected in the indexation and citation records of the journals. There is growing concern in the scientific community over the quality of research and with its visibility and citation metrics in international indexation databases. However, the editors-in-chief of Libyan journals do not seem to take journal metrics into account in their perception of their journals’ performance.

Most of the journals do not receive financial support to improve the environment and tools for managing scholarly journals, such as a journal management system and similarity check software. Indeed, lack of sufficient support was the most frequently cited challenge to journal quality improvement. But its impact can go deeper. A study on Australian journals reported that funding issues represented a major cause of journal discontinuation. ^22^ More important is that paying reviewers, combined with the widespread and relatively heavy reliance on specific reviewer committees, could affect the quality of peer review. Moreover, as the reviewer committees are associated with the journals, they are members of the journal, and it can be argued that they are internal and not external reviewers. Internal reviewers initially screen the manuscripts to check their suitability for peer review by external reviewers who are experts in the relevant fields.

Besides the inadequacy of financial resources, there is apparent inadequacy of knowledge and technical knowhow, which was alluded to by one of the responses to the question about challenges and opportunities. Browsing many journals makes this deficiency evident. A special case in point is that html versions of the articles are absent in almost all the journals. However, we stress that recruiting competent technical expertise requires adequate support from the journal owner.

The indexing in platforms such as Web of Science and Scopus for assessment of the quality of locally and regionally focused journals is not without obvious bias. While there is agreement that Clarivate Analytics’ Web of Science and Elsevier’s Scopus databases generally contain rigorously assessed, high quality journals, journals from developing countries may get rejected because their research is said to be ‘not internationally significant’ or only of ‘local interest’ to developing countries. A recent study on over 25,000 journals that use PKP’s Open Journal Systems platform found only 1.2% indexed in the Web of Science and 7.2% in Scopus. ^23^ A recent review of Pakistani journals revealed that 97% of the journals are in the lowest category and 97% do not have an impact factor. ^24^ In many developing countries, the majority of the journals are university-based. ^25^

Only one journal in our study is included in these indexes. But we should mention that we have not included two international Libyan journals because they are not published by a Libyan institution. Both are indexed in Web of Science and one has an impact factor and a CiteScore. In general, institutional academic journals are more likely to be affected by academic in-group bias and to be motivated to lower their standards for articles sharing the journal’s institutional affiliation. ^26^

Many Libyan journals are published in Arabic or both Arabic and English. Therefore, we felt it important to assess the indexation and citation of the journals in Clarivate’s ARCI index. However, there is no publicly available journal list for ARCI and information on the selection criteria is sparse. The ARCI selection criteria have been described as a “subset of the Web of Science Core Collection selection process” and are based on “traditional scientific publishing standards and the scholarly research norms of the Arab region”. ^27^

We did not search for the journals in the Arab Impact Factor (AIF) ( https://www.arabimpactfactor.com/), produced by the Association of Arab Universities, because the content is not accessible, there are no links to the journals, and there is no description of where or how citations are counted.

Indexation of Libyan journals in Google Scholar was inconsistent and incomplete, whether one is searching for individual articles or for journals as ‘source’. Technical defects in the journals are suspected. Moreover, some Libyan journals do not publish individual files of the articles but only complete issues, which prevents their indexation

In summary, we discovered defects and deficiencies that affect the performance of the journals in a large fraction of the journals, including affiliations of editors, sources of reviewers, journal management system, medium of communication with authors and reviewers, similarity check of submitted manuscripts, proof-reading of accepted manuscripts, and website organization and functionality. Moreover, the performance of the journals in terms of indexation and citation metrics was at a very low level.

Notably, there was a clear discrepancy between the high level of the editors-in-chief’s satisfaction with journal performance and the journals’ quality indicators. We do understand that lack of funding can cause deficiencies such as unavailability of similarity check software. However, it has not escaped our notice that reasons related to the desire to maintain the status quo can be hypothesized. A viewpoint article in Nature ^28^ lamented that the purpose of research publication has shifted from disseminating knowledge to “getting a publication” for the sake of promotion. Most of the journals in Libya are published by the universities, and a large portion of their academic publications are used for local faculty promotion. Thus, these journals are more likely to be motivated to lower their standards for articles written by authors who share the journal’s institutional affiliation.

Low quality publishing practices will continue to thrive as long as criteria for career advancement of university faculty members are not aligned with robust internationally accepted research performance metrics such as journal rank, citation frequencies, and H-index. We believe that raising the standard of the editorial practices and implementing more versatility and transparency in the peer review and publication process will enhance trust in the scholarly contributions of the developing world. Putting less emphasis on publications in the home journals and assigning more weight to publications in international journals may minimize the impact of academic in-group bias on the quality of the country’s scholarly publications. Yet, it is important to note that the lack of research skills, experience, and research funding is an important factor in the low-quality research in developing countries. Furthermore, in Libya as well as other developing nations, quantitative bibliometric criteria are used to assess research production for promotion decisions, regardless of the quality of publications. This could be strengthening the expedient editorial practices in many university-based journals.

One limitation in our study is that we missed several journals from a major university because all the journal websites were down for an extended period of time and the university did not respond to our inquiries. It turned out that the websites were being updated, and the information was still missing after several months during which the data were being analyzed and the manuscript was being written.

## Conclusions

The discordance between journal quality indicators and the journal editors’ satisfaction with the performance of their journals points to a broken quality system in scholarly publishing of home academic institutions. A comprehensive plan for immediate attention and subsequent action to raise the standard of academic publications, coupled with technical and training support, are needed to enhance the scientific impact in developing nations.

## Data Availability

Zenodo: Scholarly publishing in a developing nation. https://doi.org/10.5281/zenodo.8048249. ^17^ The project contains the following underlying data:
•
Underlying_data_230615.xlsx. (Anonymized survey responses). Zenodo: Scholarly publishing in a developing nation. https://doi.org/10.5281/zenodo.8048249. ^17^ This project contains the following extended data:
• SurveyMonkey_316823458.pdf. (Blank English and Arabic questionnaire used in this study). Zenodo: SRQR checklist for ‘Scholarly publishing in a developing nation’. https://doi.org/10.5281/zenodo.8048249. ^17^ Data are available under the terms of the Creative Commons Attribution 4.0 International license (CC-BY 4.0).
