# Supplementary material for: Deficient editorial practices, perceived quality, and expedient scholarly publishing in a developing nation
Source: F1000Res. 2024 Mar 21;12:1173. Originally published 2023 Sep 20. [Version 3] doi: 10.12688/f1000research.134583.3 (PMC10993009; doi:10.12688/f1000research.134583.3)
Supplement: Peer review history

# Reviewer report: Reviewer response for version 3

**DOI:** 10.5256/f1000research.163932.r288345

**Published:** 2024-06-12

**Authors:** Helen Buckley Woods

**Affiliations:** 1 Research on Research Institute and Information School, The University of Sheffield, Sheffield, England, UK

The article reports on the results of a survey and desk research exploring editorial practices in Libyan academic journals. It is an interesting article in that it presents the perspectives of editors in chief and compares them with common academic publishing quality indicators such as whether a journal is indexed in Web of Science or other bibliographic databases.

 My review is based on the qualitative aspects of the article, namely the description of methods, presentation of qualitative findings and access to source data.

 Methods

 In the methods section under ‘study design’ the article would benefit from a clear statement of what the study comprises, such as ‘This is an empirical study, consisting of a survey collecting qualitative and quantitative data, analysis of information drawn from numerous journal websites, and an amalgamation of background information from salient journal indexing and ranking systems.’ You could add subheadings (if permitted) for the descriptions of the three different approaches/methods to make this easier for the reader. You don’t mention the qualitative data and analysis until the section entitled ‘statistical analysis’. I don’t think it belongs here. The qualitative data strand needs its own section, perhaps at the end of the paragraph beginning ‘ We designed a questionnaire…’ here you could state that you have included two optional questions to capture qualitative data and why you added these questions. I think this would be helpful at this point in the article. Having introduced this qualitative strand, you could then follow this on in the rest of the article, so after the ‘statistical analysis’ section, have a ‘qualitative analysis’ section. When describing your analysis, it is not clear what you mean by ‘thematic analysis’ as this can be interpreted in many ways. It would be useful to say what the procedure was and what you mean by ‘thematic analysis’ in this instance, and give a supporting citation.

 Data availability

 I cannot find the underlying qualitative data, as the link to the anonymised survey responses doesn’t work.   

 Qualitative findings

 It would be useful to state that participants were asked two questions, namely, to state the challenges they face regarding the quality of journal publishing and how these challenges can be overcome. Then it would be beneficial to say a bit about the themes you created, and refer to your methods e.g. ‘ based on our analysis (as described in x section) we created two themes. The first theme centred on the lack of financial and institutional support provided to journal editors such as…. and then describe what the main ideas are before you give the illustrative quotations. Also, the presentation becomes a bit repetitive as you have split it into challenges and solutions, i.e. lack of support is a challenge, so we need support. I think this section could be reworked to synthesise the ideas a bit more.  

 Word cloud

 In the methods, if you add a section introducing the qualitative data collection, you could incorporate into it the paragraph beginning ‘ to enhance the trustworthiness…’ and describe what you did, how the word cloud was generated and why you decided to do this. You also need to say what it shows, for example prevalence of terms and how this links to the themes you found.

 Other comments

 Two minor points on signposting.

 In the abstract ‘conclusions’ section you say ‘similar expedient publishing practices might exist in other countries as well’ it would be good to say why your results lead you to this conclusion, or just take this sentence out.

 In the ‘website based journal assessment’ section you could state what you present after the first paragraph and before launching into the list. This is at the top of page 10, you could add ‘in this section we list xyz’

Is the work clearly and accurately presented and does it cite the current literature?

Partly

If applicable, is the statistical analysis and its interpretation appropriate?

Not applicable

Are all the source data underlying the results available to ensure full reproducibility?

Partly

Is the study design appropriate and is the work technically sound?

Yes

Are the conclusions drawn adequately supported by the results?

No

Are sufficient details of methods and analysis provided to allow replication by others?

No

Reviewer Expertise:

NA

I confirm that I have read this submission and believe that I have an appropriate level of expertise to confirm that it is of an acceptable scientific standard, however I have significant reservations, as outlined above.

# Reviewer report: Reviewer response for version 3

**DOI:** 10.5256/f1000research.163932.r258063

**Published:** 2024-04-03

**Authors:** Andreas Nishikawa-Pacher

**Affiliations:** 1 Department of Legal and Constitutional History, University of Vienna, Vienna, Vienna, Austria; 2 Diplomatic Academy Vienna, Vienna, Vienna, Austria; 3 Bibliothek, Technische Universitat Wien, Vienna, Vienna, Austria

The two comments I had in my last report were appropriately addressed. The two comments I had in my last report were appropriately addressed. First, the authors added a (more easily machine-readable) version of the dataset; and second, they removed some incorrect statements.

Is the work clearly and accurately presented and does it cite the current literature?

Partly

If applicable, is the statistical analysis and its interpretation appropriate?

I cannot comment. A qualified statistician is required.

Are all the source data underlying the results available to ensure full reproducibility?

Partly

Is the study design appropriate and is the work technically sound?

Partly

Are the conclusions drawn adequately supported by the results?

Yes

Are sufficient details of methods and analysis provided to allow replication by others?

Partly

Reviewer Expertise:

Meta-scientific infrastructures

I confirm that I have read this submission and believe that I have an appropriate level of expertise to confirm that it is of an acceptable scientific standard.

# Reviewer report: Reviewer response for version 3

**DOI:** 10.5256/f1000research.163932.r258064

**Published:** 2024-03-23

**Authors:** Hamid Jamali

**Affiliations:** 1 Charles Sturt University, Wagga Wagga, NSW, Australia

The revision has improved the paper.

Is the work clearly and accurately presented and does it cite the current literature?

Partly

If applicable, is the statistical analysis and its interpretation appropriate?

Yes

Are all the source data underlying the results available to ensure full reproducibility?

Yes

Is the study design appropriate and is the work technically sound?

Partly

Are the conclusions drawn adequately supported by the results?

Yes

Are sufficient details of methods and analysis provided to allow replication by others?

Partly

Reviewer Expertise:

Scholarly communication

I confirm that I have read this submission and believe that I have an appropriate level of expertise to confirm that it is of an acceptable scientific standard.

# Reviewer report: Reviewer response for version 2

**DOI:** 10.5256/f1000research.159108.r227208

**Published:** 2024-03-13

**Authors:** Andreas Nishikawa-Pacher

**Affiliations:** 1 Department of Legal and Constitutional History, University of Vienna, Vienna, Vienna, Austria; 2 Diplomatic Academy Vienna, Vienna, Vienna, Austria; 3 Bibliothek, Technische Universitat Wien, Vienna, Vienna, Austria

Thanks for the revision, the manuscript remains interesting as before, and some of the additions have certainly enhanced the way the article is embedded into the relevant literature.

- The new data at Zenodo ( https://zenodo.org/doi/10.5281/zenodo.10155317) should also be included under "Data availability". I think it would have been better if the data were in a machine-readable format (like CSV) rather than a PDF, but given the small number of journals, I guess having a PDF is not too cumbersome. At least the sample is made transparent now.
- As regards the addition about CrossRef, I think you can omit the reference to OpenAlex or LENS; it is, to my knowledge, factually wrong to state that journals are in CrossRef but not in the either of the two other sources, since everything deposited in CrossRef is also in the sample of OpenAlex and LENS. For example, journal #2 (according to the dataset at Zenodo) is in CrossRef according to https://api.crossref.org/journals/2664-1674 but also in OpenAlex according to https://api.openalex.org/sources?filter=issn:2664-1674. But the issue is so minor that I would suggest deleting the mentioning of OpenAlex and LENS, so as to not confuse others (sorry if my comment in my 1st review was too advanced in this matter, thus deviating from the main focus of the manuscript).

Is the work clearly and accurately presented and does it cite the current literature?

Partly

If applicable, is the statistical analysis and its interpretation appropriate?

I cannot comment. A qualified statistician is required.

Are all the source data underlying the results available to ensure full reproducibility?

Partly

Is the study design appropriate and is the work technically sound?

Partly

Are the conclusions drawn adequately supported by the results?

Yes

Are sufficient details of methods and analysis provided to allow replication by others?

Partly

Reviewer Expertise:

Meta-scientific infrastructures

I confirm that I have read this submission and believe that I have an appropriate level of expertise to confirm that it is of an acceptable scientific standard, however I have significant reservations, as outlined above.

# Response

**Published:** 2024-03-16

**Authors:** Omran Bakoush

**Affiliations:** Internal Medicine, United Arab Emirates University, Alain, AbuDhabi, United Arab Emirates

**RESPONSES TO REVIEWER: Andreas Nishikawa-Pacher**

 Comment 1: The new data at Zenodo (https://zenodo.org/doi/10.5281/zenodo.10155317) should also be included under "Data availability". I think it would have been better if the data were in a machine-readable format (like CSV) rather than a PDF, but given the small number of journals, I guess having a PDF is not too cumbersome. At least the sample is made transparent now."

 RESPONSE: We thank the reviewer for his attention to detail. We have uploaded an Excel file of the journals' list and updated the Data Availability section with the information and the link.

 Comment 2: As regards the addition about CrossRef, I think you can omit the reference to OpenAlex or LENS; it is, to my knowledge, factually wrong to state that journals are in CrossRef but not in the either of the two other sources, since everything deposited in CrossRef is also in the sample of OpenAlex and LENS. For example, journal #2 (according to the dataset at Zenodo) is in CrossRef according to https://api.crossref.org/journals/2664-1674 but also in OpenAlex according to https://api.openalex.org/sources?filter=issn:2664-1674. But the issue is so minor that I would suggest deleting the mentioning of OpenAlex and LENS, so as to not confuse others (sorry if my comment in my 1st review was too advanced in this matter, thus deviating from the main focus of the manuscript).

 RESPONSE: We thank the reviewer for this important comment. We have deleted mention of OpenAlex and LENS.

# Reviewer report: Reviewer response for version 2

**DOI:** 10.5256/f1000research.159108.r227209

**Published:** 2024-02-05

**Authors:** Hamid Jamali

**Affiliations:** 1 Charles Sturt University, Wagga Wagga, NSW, Australia

I thank the authors for thoroughly addressing the reviewers' comments. I believe the revised version of the paper is much-improved and suitable for indexing. The article contributes well to our understanding of scholarly publishing in developing countries, particularly in the Arab region.

Is the work clearly and accurately presented and does it cite the current literature?

Partly

If applicable, is the statistical analysis and its interpretation appropriate?

Yes

Are all the source data underlying the results available to ensure full reproducibility?

Yes

Is the study design appropriate and is the work technically sound?

Partly

Are the conclusions drawn adequately supported by the results?

Yes

Are sufficient details of methods and analysis provided to allow replication by others?

Partly

Reviewer Expertise:

Scholarly communication

I confirm that I have read this submission and believe that I have an appropriate level of expertise to confirm that it is of an acceptable scientific standard.

# Reviewer report: Reviewer response for version 1

**DOI:** 10.5256/f1000research.147648.r216185

**Published:** 2023-11-03

**Authors:** Andreas Nishikawa-Pacher

**Affiliations:** 1 Department of Legal and Constitutional History, University of Vienna, Vienna, Vienna, Austria; 2 Diplomatic Academy Vienna, Vienna, Vienna, Austria; 3 Bibliothek, Technische Universitat Wien, Vienna, Vienna, Austria

Thanks for inviting me to review the paper on Libyan journals performances and the perceptions of their editors regarding the journals' quality. It offers an interesting read, with some fascinating insights that complement the conventional knowledge about the scientific publication system. Conventionally, meta-scientific analyses tend to focus on journals that are indexed in Web of Science or Scopus, and that are published in English language and that conform to the so-called 'Global North' standards; this manuscript, in contrast, investigated an aspect of the scholarly publication system within a rather local setting in Libya.

 A key insight is that there is a discrepancy between the editors' own assessment of their journals' performances on the one hand, and more or less 'objective' indicators on the other hand. What is also of interest are some of the "anomalies" (when compared to the usual convention), such as the fact that one journal pursues the editorial processing only via personal contact (rather than an online submission system or via e-mail), or that some of the journals lack a professional online presentation (e.g., websites with bogus "Impact Factors", or journal websites that only offer PDF versions rather than HTML versions of the texts, with too-many clicks necessary to access the scientific content). It is also remarkable that 80% of the journals seem to be open access and operate free of charge (i.e., Diamond Open Access, albeit not necessarily part of DOAJ).

 The introduction is well written, and the manuscript structure conforms to the usual IMRaD-structure. However, I believe the paper would gain in strength with some additional information.

 First, given that readers (like me) may not be an expert in the methodical approach used here, it would be great if the authors at least partly referenced the general standards of such expert surveys and quantitative interviews, and to show how their own approach conforms to these standards.

 Second, is it possible to offer at least the list of journals that are covered by this study? The authors do not have to identify the responses (available at Zenodo) with the journals, but just having a catalogue of the journal sample would be interesting.

 Third, I think the focus on Web of Science, Scopus and Google Scholar merits a complementary view with similar (but 'emerging') databases, such as Dimensions, or, more importantly, the "open data"-based CrossRef, LENS, and OpenAlex. The journal coverages differ from each other, and from my knowledge, the latter three or four databases are likewise widely used. And if readers had knowledge about the journals being members of, say, CrossRef, then more data could be fetched via CrossRef metadata.

 Fourth, did the authors further delve into some of the concerning matters, such as the fact that bogus impact factors were displayed? Is there anything to say with regards to "predatory" publication structures that may be at play behind some journals? Why does a website present such questionable metrics -- is it an innocent lack of knowledge, or is there a deceiving intent behind it? Would an expert survey even be able to unearth a "predatory" practice, given that respondents may not be honest in their responses? Another issue worthwhile to be addressed would be whether the journals use PIDs (especially DOIs), and if so, what kind of metadata are deposited behind the DOIs.

 Fifth, the whole manuscript revolves around analyzing Libyan journals against a global standard -- in the Discussion section, could anything be stated, at least briefly, about this "imposition" of common norms upon the Libyan system? Is there some kind of incompatibility between the global norms and the local practice? The wording of  "colonialism" may be an overstretch here, but at least I got the impression that some readers may be interested in hearing more about this concept in this context.

 Finally, there are some minor inconsistencies or errors, such as "DOAJ" being abbreviated as "DOJA", or that the abbreviation of "EC" was introduced for editors-in-chief, but then "EIC" is used later, etc. These are just minor matters.

 As a further, very general remark, it seemed to me that the paper could better integrate current research on similar topics, at least briefly, so as to embed their own investigation into the relevant literature. Having just 10 references to peer-reviewed works does not really do justice to the fact that a lot is being researched on similar meta-scientific issues regarding the scientific journal landscape (e.g., on AJOL ["African Journals Online] ^1^, on journals that publish in LOTE [languages other than English] ^2^, on possibly "predatory" practices ^3^, or, to cite merely one of our own examples ^4^, whether a journal really attains the status of "open access" if the website poses even minor technical barriers in accessing the content (e.g., no HTML but only PDF versions).

 Despite these few remarks on how the manuscript could possibly enhance its quality, the paper already offers a novel study with interesting insights for science at large.

Is the work clearly and accurately presented and does it cite the current literature?

Partly

If applicable, is the statistical analysis and its interpretation appropriate?

I cannot comment. A qualified statistician is required.

Are all the source data underlying the results available to ensure full reproducibility?

Partly

Is the study design appropriate and is the work technically sound?

Partly

Are the conclusions drawn adequately supported by the results?

Yes

Are sufficient details of methods and analysis provided to allow replication by others?

Partly

Reviewer Expertise:

Meta-scientific infrastructures

I confirm that I have read this submission and believe that I have an appropriate level of expertise to confirm that it is of an acceptable scientific standard, however I have significant reservations, as outlined above.

## References

[1] : Is it open access if registration is required to obtain scientific content?. *European Science Editing* .2023;49: 10.3897/ese.2023.e98101 10.3897/ese.2023.e98101

[2] : African Journals Online: improving awareness and access. *Learned Publishing* .2002;15(1) : 10.1087/095315102753303689 51-57 10.1087/095315102753303689

[3] : A walk on the wild side: ‘Predatory’ journals and information asymmetries in scientific evaluations. *Research Policy* .2019;48(2) : 10.1016/j.respol.2018.04.013 462-477 10.1016/j.respol.2018.04.013

[4] : Publish (in English) or perish: The effect on citation rate of using languages other than English in scientific publications. *Ambio* .2017;46(1) : 10.1007/s13280-016-0820-7 121-127 10.1007/s13280-016-0820-7 27686730 PMC5226904

# Response

**Published:** 2023-11-18

**Authors:** Omran Bakoush

**Affiliations:** Internal Medicine, United Arab Emirates University, Alain, AbuDhabi, United Arab Emirates

**Response to Reviewer Report**

 Reviewer-2: Andreas Nishikawa-Pacher, Diplomatic Academy Vienna, Vienna, Vienna, Austria; 

 We thank reviewer for reviewing our manuscript and describing our study as novel and insightful. We also appreciate his comments, which enabled us to add value to our manuscript.

**Point by point responses**

**Review comment:**

 First, given that readers (like me) may not be an expert in the methodical approach used here, it would be great if the authors at least partly referenced the general standards of such expert surveys and quantitative interviews, and to show how their own approach conforms to these standards.

**Authors Reply:**

 We based the questionnaire on the editorial policies published by the Council of Science Editors and on the Web of Science basic journal selection criteria for indexation. We augmented the description of questionnaire design as follows "We designed a questionnaire consisting of 10 parts following the best practices in designing questionnaire items and organizing the questionnaire. ^"^

 The questionnaire was pre-tested by the researchers and then piloted online by three editors of an international journal who were not affiliated with a local or regional institution. It was ensured that the logic of the online questionnaire functioned correctly, and any ambiguous or misunderstood questions were re-worded.

 23. Gehlbach, H., & Brinkworth, M. E. (2011). Measure twice, cut down error: A process for enhancing the validity of survey scales. Review of General Psychology, 15(4), 380–387 (PMID: 29210756).

 24. Gehlbach H, Artino AR Jr. The Survey Checklist (Manifesto). Acad Med. 2018 Mar;93(3):360-366. doi: 10.1097/ACM.0000000000002083. PMID: 29210756.

**Review comment:**

 Second, is it possible to offer at least the list of journals that are covered by this study? The authors do not have to identify the responses (available at Zenodo) with the journals, but just having a catalogue of the journal sample would be interesting.

**Authors Reply:**

 We uploaded a list of the journals included in the study to a repository:

 Therefore, the study included 48 academic journals published by national institutions (list available at https:// 10.5281/zenodo.10155318).

**Review comment:**

 Third, I think the focus on Web of Science, Scopus and Google Scholar merits a complementary view with similar (but 'emerging') databases, such as Dimensions, or, more importantly, the "open data"-based CrossRef, LENS, and OpenAlex. The journal coverages differ from each other, and from my knowledge, the latter three or four databases are likewise widely used. And if readers had knowledge about the journals being members of, say, CrossRef, then more data could be fetched via CrossRef metadata.

**Authors Reply:**

 We have now checked for indexation of the journals in the Arab abstract-indexing database, eMarefa, and added the following:

 "On the other hand, Marefa (e-Marefa.net) is an abstract-indexing database established in 2008 for peer-reviewed literature from Arab countries published in Arabic, English or French. It indexes over 4000 journals and is the basis of the Arab Citation & Impact Factor (ARCIF, https://emarefa.net/ARCIF/). Of the 48 journals included in this study, 13 (27%) are listed in e-Marefa.com, with ARCIF factors ranging from zero to 1.3. **"**

 "We did not search for the journals in the Arab Impact Factor (AIF) (https://www.arabimpactfactor.com/), produced by the Association of Arab Universities, because the content is not accessible, there are no links to the journals, and there is no description of where or how citations are counted."

**Review comment:**

 Fourth, did the authors further delve into some of the concerning matters, such as the fact that bogus impact factors were displayed? Is there anything to say with regards to "predatory" publication structures that may be at play behind some journals? Why does a website present such questionable metrics -- is it an innocent lack of knowledge, or is there a deceiving intent behind it? Would an expert survey even be able to unearth a "predatory" practice, given that respondents may not be honest in their responses? Another issue worthwhile to be addressed would be whether the journals use PIDs (especially DOIs), and if so, what kind of metadata are deposited behind the DOIs.

**Authors Reply:**

 The relevant text now reads as follows:

 Notably, a few journals advertised on their websites impact factors that are known as or suspected of being fake. Advertising a questionable impact factor can be detrimental to a journal, and any claim to having an impact factor should specify its source. When the source of a published impact factor is not specified, a deceiving intent to provide false impression about journal status is suspected.

**Review comment:**

 Fifth, the whole manuscript revolves around analyzing Libyan journals against a global standard -- in the Discussion section, could anything be stated, at least briefly, about this "imposition" of common norms upon the Libyan system? Is there some kind of incompatibility between the global norms and the local practice? The wording of "colonialism" may be an overstretch here, but at least I got the impression that some readers may be interested in hearing more about this concept in this context.

**Authors Reply:**

 We added the following in the discussion:

 Yet, it is important to note that the lack of research skills, experience, and research funding is an important factor in the low-quality research in developing countries. Furthermore, in Libya as well as other developing nations, quantitative bibliometric criteria are used to assess research production for promotion decisions, regardless of the quality of publications. This could be strengthening the expedient editorial practices in many university-based journals

**Review comment:**

 Finally, there are some minor inconsistencies or errors, such as "DOAJ" being abbreviated as "DOJA", or that the abbreviation of "EC" was introduced for editors-in-chief, but then "EIC" is used later, etc. These are just minor matters.

**Authors Reply:**

 Done. Thank you.   

**Review comment:**

 As a further, very general remark, it seemed to me that the paper could better integrate current research on similar topics, at least briefly, so as to embed their own investigation into the relevant literature. Having just 10 references to peer-reviewed works does not really do justice to the fact that a lot is being researched on similar meta-scientific issues regarding the scientific journal landscape (e.g., on AJOL ["African Journals Online]1, on journals that publish in LOTE [languages other than English]2, on possibly "predatory" practices3, or, to cite merely one of our own examples4, whether a journal really attains the status of "open access" if the website poses even minor technical barriers in accessing the content (e.g., no HTML but only PDF versions).

**Authors response**

 We have provided more information from nine more references.

# Reviewer report: Reviewer response for version 1

**DOI:** 10.5256/f1000research.147648.r207959

**Published:** 2023-11-03

**Authors:** Hamid Jamali

**Affiliations:** 1 Charles Sturt University, Wagga Wagga, NSW, Australia

The article illustrates a picture of scholarly journal publishing in Libya by presenting the results of a survey of journal editors and some analysis of data collected through desk research from journals' websites. The results are mainly related to 48 journals and their editors who responded to the questionnaire. 

 It is helpful to know about the status quo of journal publishing in a developing country and the challenges faced by those journals and in this regard, the paper is useful. However, I found the analysis a bit too descriptive and there is some room for improvement.

 To start with, I think the paper needs to provide some background about journal publishing in Libya as the context. When did journal publishing start, how many journals exist, in what language are they published and so on? Some information about the country would also be useful as context (population, number of universities, number of papers published by Libyan researchers in Scopus in one year as an example etc). This information should be available somewhere or at least collectable via some desk research. The authors have relied on the questionnaire to find some facts about journals while I believe that type of information could be more accurately collected via desk research (e.g. indexation, language and so on). The initial analysis should not be limited only to those journals that completed the questionnaire, it should cover all Libyan journals if possible. 

 The survey results are presented appropriately (simple frequency and percentage) but I expected a bit more at least from free-text questions. The word cloud doesn't reveal much really as it only includes a few general terms such as journal, institution etc. So the authors might see if they can improve the analysis to add more depth and insight. 

 The paper can benefit from better coverage of the relevant literature. I have provided a few references here ^1^^-^[ref-6. Some are examples of papers that discuss journal publishing in individual developing countries (Pakistan and Nigeria), and some are discussions particularly about journal publishing in the Arab world (which Libya is part of). One is generally about journal publishing in the world (Khanna et al) which has good points and information about local journals and non-English journals. And finally, Jamali et al (disclaimer: my own paper on local journals in Australia) has information about challenges faced by local journals that occasionally result in their discontinuation.

 The discussion has good points, but you can also consider the value of local/national journals in your discussion. Local/national journals have functions that can be distinct from international journals.

Is the work clearly and accurately presented and does it cite the current literature?

Partly

If applicable, is the statistical analysis and its interpretation appropriate?

Yes

Are all the source data underlying the results available to ensure full reproducibility?

Yes

Is the study design appropriate and is the work technically sound?

Partly

Are the conclusions drawn adequately supported by the results?

Yes

Are sufficient details of methods and analysis provided to allow replication by others?

Partly

Reviewer Expertise:

Scholarly communication

I confirm that I have read this submission and believe that I have an appropriate level of expertise to confirm that it is of an acceptable scientific standard, however I have significant reservations, as outlined above.

## References

[1] : Recalibrating the scope of scholarly publishing: A modest step in a vast decolonization process. *Quantitative Science Studies* .2022;3(4) : 10.1162/qss_a_00228 912-930 10.1162/qss_a_00228

[2] : Why do journals discontinue? A study of Australian ceased journals. *Learned Publishing* .2022;35(2) : 10.1002/leap.1448 219-228 10.1002/leap.1448

[3] : Challenges in Journal Publishing in the Arab World. *Journal of Information Studies & Technology (JIS&T)* .2018;2018(1) : 10.5339/jist.2018.1 10.5339/jist.2018.1

[4] : Biomedical Journal Publishing in Yemen: Challenges and Prospects. *University of Science and Technology Journal for Medical Sciences* .2023;1(1) : 10.59222/ustjms.1.1.E 10.59222/ustjms.1.1.E

[5] : Giant in Isolation: Online Journal Publishing in Nigeria. *The Journal of Electronic Publishing* .2023;26(1) : 10.3998/jep.2640 10.3998/jep.2640

[6] : Status of open access scholarly journal publishing in Pakistan. *Journal of Librarianship and Information Science* .2023; 10.1177/09610006221146301 10.1177/09610006221146301

# Response

**Published:** 2023-11-18

**Authors:** Omran Bakoush

**Affiliations:** Internal Medicine, United Arab Emirates University, Alain, AbuDhabi, United Arab Emirates

**Response to Reviewer Reports**

 Reviewer-1: Hamid Jamali, Charles Sturt University, Wagga Wagga, NSW, Australia

 We would like to thank Reviewers for taking the necessary time and effort to review the manuscript. We sincerely appreciate all your valuable comments and suggestions, which helped us in improving the quality of the manuscript.

**Point by point responses**

**Review comment:**

 It is helpful to know about the status quo of journal publishing in a developing country and the challenges faced by those journals and in this regard, the paper is useful. However, I found the analysis a bit too descriptive and there is some room for improvement.

**Authors' Reply:**

 Although the analysis design of the study was primarily descriptive, we elaborated on the findings using qualitative analysis of respondents' responses to open questions on challenges facing their journals. Further, we used the data generated from desk research on journal quality to elaborate on the survey responses. The mixed methods approach was used to integrate insights gained from the survey, desk research data, and open responses, which was helpful in enhancing the validity of the interpretations.

**Review comment:**

 To start with, I think the paper needs to provide some background about journal publishing in Libya as the context. When did journal publishing start, how many journals exist, in what language are they published and so on? Some information about the country would also be useful as context (population, number of universities, number of papers published by Libyan researchers in Scopus in one year as an example etc). This information should be available somewhere or at least collectable via some desk research.

**Authors' Reply:**

 To comply with the reviewer's request, we have added the following in the introduction:

 Libya is an Arab country with an estimated surface area of 1,775,500 km ^2^ and a population of about 6.7 million. Most of the population live in the main coastal cities, namely Tripoli (the capital) and Benghazi (second largest city). Since the start of oil exportation from Libya in the 1960s, living standards have risen. Undergraduate and postgraduate institutions have been expanded and improved over the years. The first Libyan university was established in 1957 (https://mhesr.gov.ly/?page_id=126). The number of universities has expanded over time, and according to the ministry of education, there are now 26 public universities in Libya with 350,000 university students. ^20^

 The first initiative to formally promote research in Libya began in the mid-seventies with the establishment of the National Commission of Scientific Research, formally charged with planning and supporting scientific research. Over many years, the Libyan government, expanded the scope of MSc degree programs, and recently initiated PhD programs in various fields, including sciences, as well as built research centers. However, overall research performance indicators remain modest.

 The Libyan Journal of Science, the first scholarly journal published in Libya, was launched in 1971. It is improbable that it is the currently active journal by the same name because the numbering of the annual volumes goes back only to the late nineties. Jamahiriya Medical Journal was launched in 1976 and continues to be listed in Scopus (without metrics) though it ceased its activity in 2011. A recent bibliometric study (Jaradat) ^21^ based on the Scopus database for Libya's scientific output from 1948 to 2022 revealed a total number of 10,475 articles in the Scopus database, 10361 of them in English (99%).  On the other hand, another study (Mahmood) ^22^, based on the Directory and Book of Abstracts of Libya’s National Centre for Scientific Research for 2001-2004, revealed that 45% of the research output was reported in English, and the rest was in Arabic.

**Review comment:**

 The authors have relied on the questionnaire to find some facts about journals while I believe that type of information could be more accurately collected via desk research (e.g. indexation, language and so on). The initial analysis should not be limited only to those journals that completed the questionnaire, it should cover all Libyan journals if possible.

**Authors' Reply:**

 We collected data about editorial journal qualities from the websites only of the journals the editors of which participated in the study. The aim was to integrate the data from the questionnaire with those from desk searches for the journal editorial qualities. This yielded perhaps the most notable finding of the discrepancy between the editors-in-chiefs' perception of journal performance and objective criteria of journal editorial performance. Furthermore, most of the other journals either did not have publishing websites or were not active at the time of the search. The relevant section of the methods has been modified slightly and augmented as follows:

 To integrate results of desk research with the results from the questionnaire, the websites of the journals the editors of which returned questionnaire responses were visited by one investigator (AB) and information was collected on various journal qualities. These included but were not limited to the presence of an International Standard Serial Number (ISSN), the editorial board's geographic diversity, the organization of the archives, and general website functionality. Moreover, data were collected on the listing of the journals in Google Scholar and the number of citations of all articles published in 2019, as well as indexation in the database of the Arab Citation & Impact Factor (Arcif).

**Review comment:**

 The survey results are presented appropriately (simple frequency and percentage) but I expected a bit more at least from free-text questions. The word cloud doesn't reveal much really as it only includes a few general terms such as journal, institution etc. So the authors might see if they can improve the analysis to add more depth and insight.

**Authors Reply:**

 "Financial support" is one the three most prominent terms in the word cloud. Nevertheless, we replaced the figure with a new figure that includes more words and thus gives a clearer picture.

**Review comment:**

 The paper can benefit from better coverage of the relevant literature. I have provided a few references here1-[ref-6. Some are examples of papers that discuss journal publishing in individual developing countries (Pakistan and Nigeria), and some are discussions particularly about journal publishing in the Arab world (which Libya is part of). One is generally about journal publishing in the world (Khanna et al) which has good points and information about local journals and non-English journals. And finally, Jamali et al (disclaimer: my own paper on local journals in Australia) has information about challenges faced by local journals that occasionally result in their discontinuation.

**Authors Reply:**

 We thank the reviewer for suggesting the references. We have added the following in the discussion:

 "A recent study on over 25,000 journals that use PKP's Open Journal Systems platform found only 1.2% indexed in the Web of Science and 7.2% in Scopus.   A recent review of Pakistani journals revealed that 97% of the journals are in the lowest category and 97% do not have an impact factor. ^26^ In many developing countries, the majority of the journals are university-based."

 We also added the following at an appropriate place in the discussion: But its impact can go deeper. A study on Australian journals reported that funding issues represented a major cause of journal discontinuation. ^28^

**Review comment:**

 The discussion has good points, but you can also consider the value of local/national journals in your discussion. Local/national journals have functions that can be distinct from international journals.

**Authors Reply:**

 We have added the following text in the discussion:

 However, local journals should not be undervalued. Researchers might prefer to publish in international journals, but local journals have distinct, useful functions. Whereas international journals provide a platform for researchers to share their research globally, local journals provide a platform for issues and developments relevant to the local community that might not be considered relevant to international journals. Local journals also foster cooperation between local researchers and provide a platform for publishing in their native language. Nevertheless, there is a need for establishing robust criteria for publishing practices and policies of local journals, particularly state-sponsored journals.
